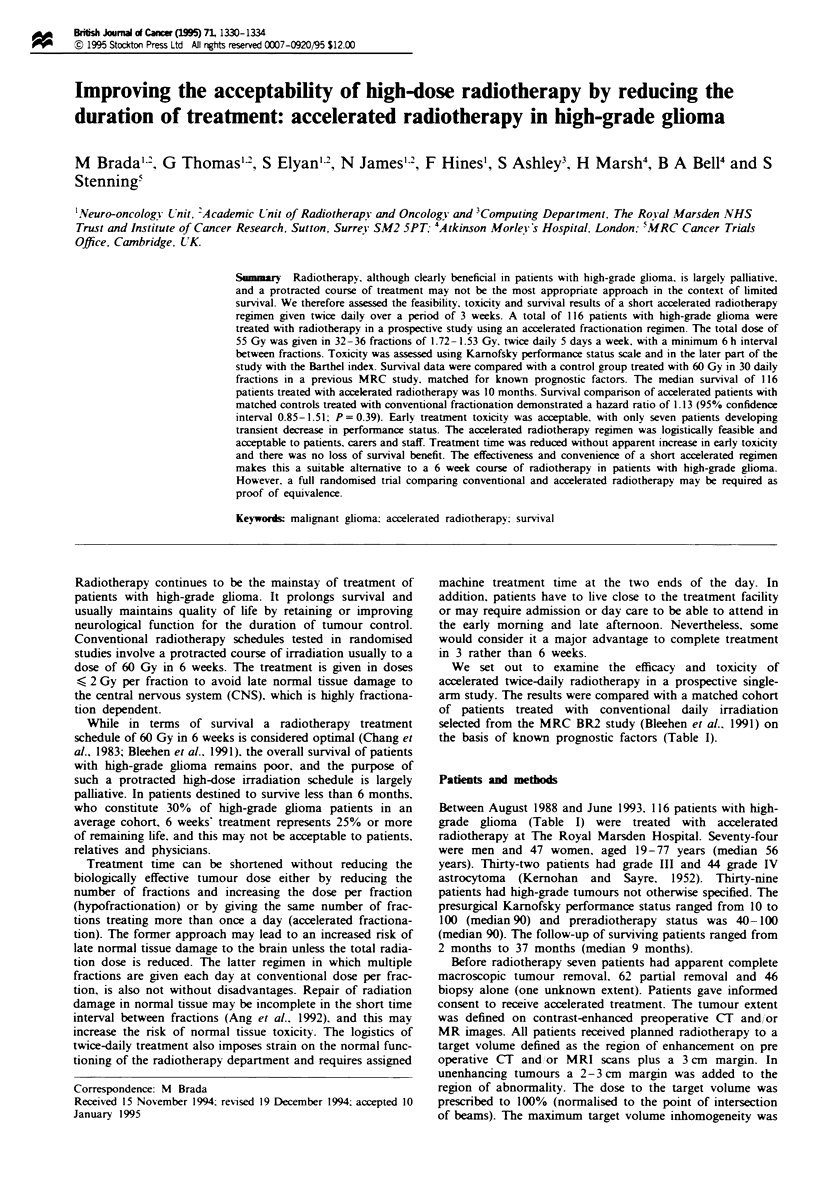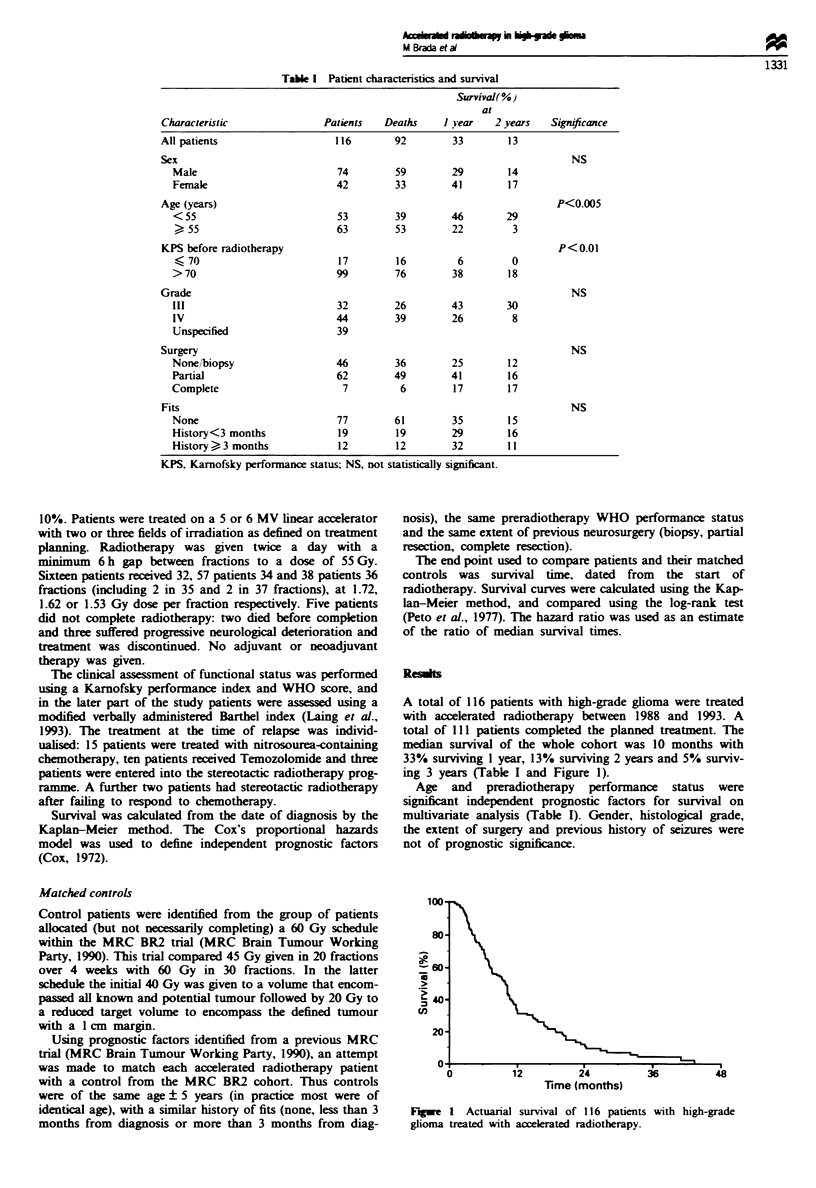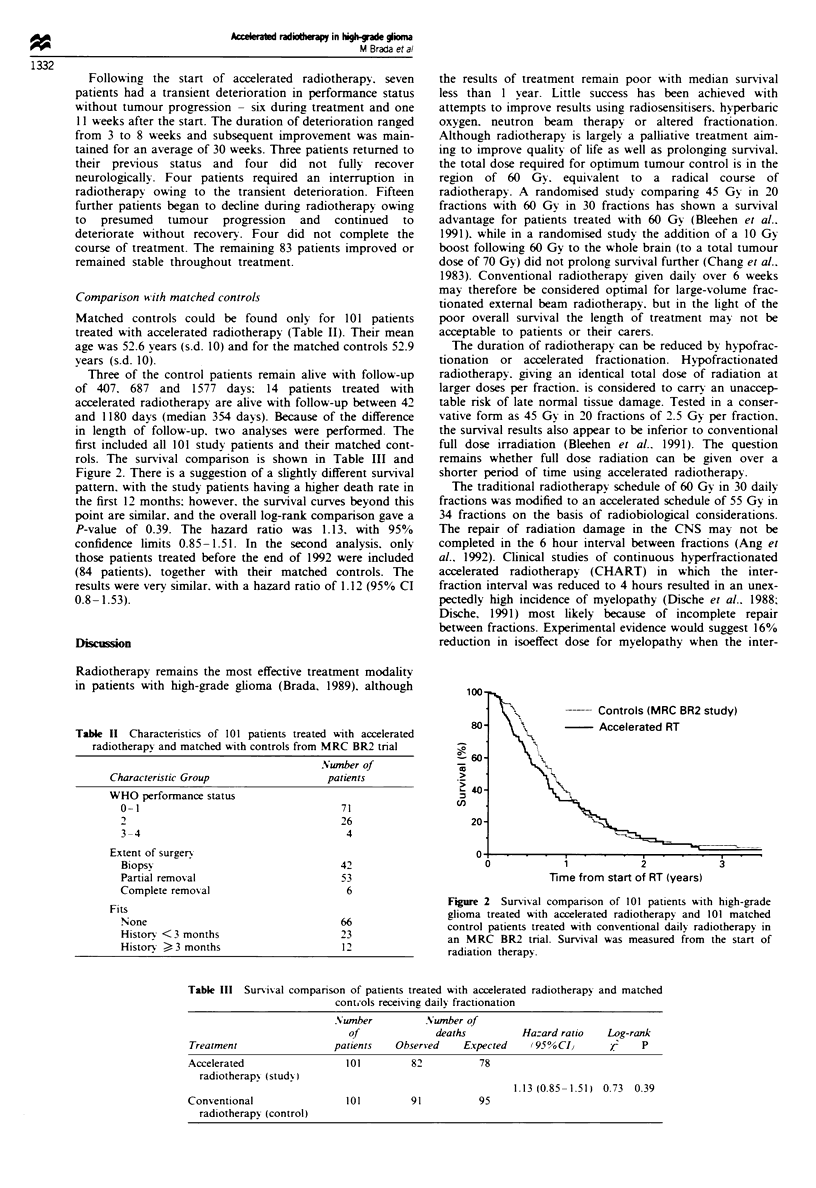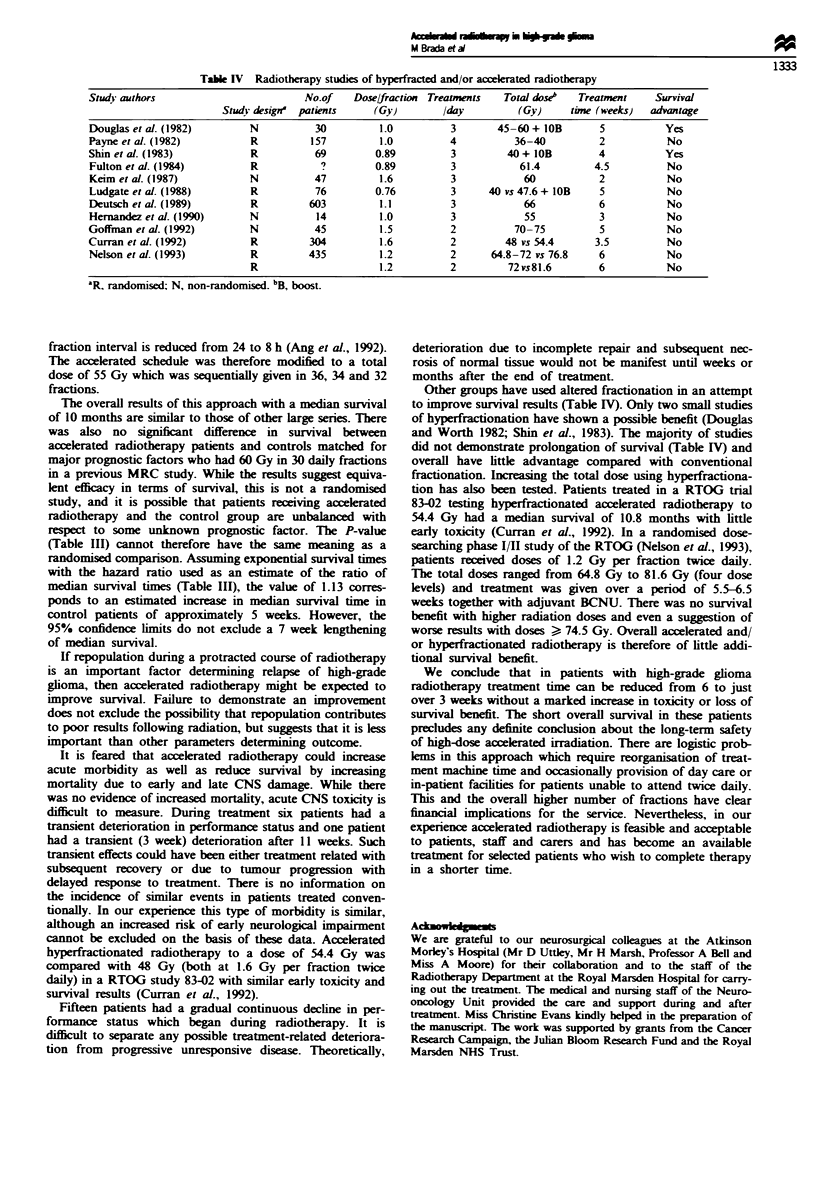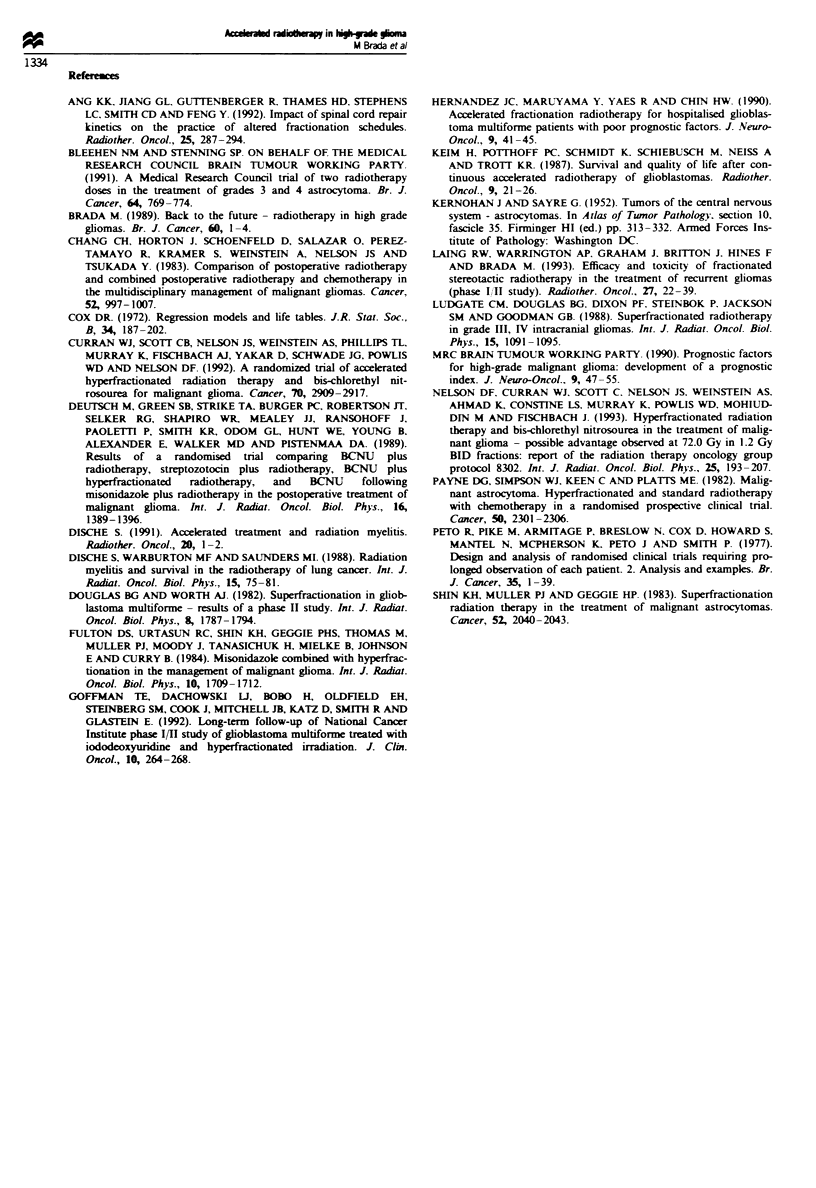# Improving the acceptability of high-dose radiotherapy by reducing the duration of treatment: accelerated radiotherapy in high-grade glioma.

**DOI:** 10.1038/bjc.1995.258

**Published:** 1995-06

**Authors:** M. Brada, G. Thomas, S. Elyan, N. James, F. Hines, S. Ashley, H. Marsh, B. A. Bell, S. Stenning

**Affiliations:** Neuro-oncology Unit, Royal Marsden NHS Trust, Sutton, Surrey, UK.

## Abstract

Radiotherapy, although clearly beneficial in patients with high-grade glioma, is largely palliative, and a protracted course of treatment may not be the most appropriate approach in the context of limited survival. We therefore assessed the feasibility, toxicity and survival results of a short accelerated radiotherapy regimen given twice daily over a period of 3 weeks. A total of 116 patients with high-grade glioma were treated with radiotherapy in a prospective study using an accelerated fractionation regimen. The total dose of 55 Gy was given in 32-36 fractions of 1.72-1.53 Gy, twice daily 5 days a week, with a minimum 6 h interval between fractions. Toxicity was assessed using Karnofsky performance status scale and in the later part of the study with the Barthel index. Survival data were compared with a control group treated with 60 Gy in 30 daily fractions in a previous MRC study, matched for known prognostic factors. The median survival of 116 patients treated with accelerated radiotherapy was 10 months. Survival comparison of accelerated patients with matched controls treated with conventional fractionation demonstrated a hazard ratio of 1.13 (95% confidence interval 0.85-1.51; P = 0.39). Early treatment toxicity was acceptable, with only seven patients developing transient decrease in performance status. The accelerated radiotherapy regimen was logistically feasible and acceptable to patients, carers and staff. Treatment time was reduced without apparent increase in early toxicity and there was no loss of survival benefit. The effectiveness and convenience of a short accelerated regimen makes this a suitable alternative to a 6 week course of radiotherapy in patients with high-grade glioma. However, a full randomised trial comparing conventional and accelerated radiotherapy may be required as proof of equivalence.


© 1995 Stockton Press Ltd All rights reserved 0007-0920/95 $12.00

### Improving the acceptability of high-dose radiotherapy by reducing the duration of treatment: accelerated radiotherapy in high-grade glioma

M Brada[1,2], G Thomas[1,2], S Elyan[1,2], N James[1,2], F Hines[1], S Ashley[3], H Marsh[4], B A Bell[4] and S Stenning[5]

[1]*Neuro-oncology Unit,* [2]*Academic Unit of Radiotherapy and Oncology and* [3]*Computing Department, The Royal Marsden NHS Trust and Institute of Cancer Research, Sutton, Surrey SM2 5PT;* [4]*Atkinson Morley's Hospital, London;* [5]*MRC Cancer Trials Office, Cambridge, UK.*

**Summary** Radiotherapy, although clearly beneficial in patients with high-grade glioma, is largely palliative, and a protracted course of treatment may not be the most appropriate approach in the context of limited survival. We therefore assessed the feasibility, toxicity and survival results of a short accelerated radiotherapy regimen given twice daily over a period of 3 weeks. A total of 116 patients with high-grade glioma were treated with radiotherapy in a prospective study using an accelerated fractionation regimen. The total dose of 55 Gy was given in 32-36 fractions of 1.72-1.53 Gy, twice daily 5 days a week, with a minimum 6 h interval between fractions. Toxicity was assessed using Karnofsky performance status scale and in the later part of the study with the Barthel index. Survival data were compared with a control group treated with 60 Gy in 30 daily fractions in a previous MRC study, matched for known prognostic factors. The median survival of 116 patients treated with accelerated radiotherapy was 10 months. Survival comparison of accelerated patients with matched controls treated with conventional fractionation demonstrated a hazard ratio of 1.13 (95% confidence interval 0.85-1.51; $P = 0.39$). Early treatment toxicity was acceptable, with only seven patients developing transient decrease in performance status. The accelerated radiotherapy regimen was logistically feasible and acceptable to patients, carers and staff. Treatment time was reduced without apparent increase in early toxicity and there was no loss of survival benefit. The effectiveness and convenience of a short accelerated regimen makes this a suitable alternative to a 6 week course of radiotherapy in patients with high-grade glioma. However, a full randomised trial comparing conventional and accelerated radiotherapy may be required as proof of equivalence.

**Keywords:** malignant glioma; accelerated radiotherapy; survival

Radiotherapy continues to be the mainstay of treatment of patients with high-grade glioma. It prolongs survival and usually maintains quality of life by retaining or improving neurological function for the duration of tumour control. Conventional radiotherapy schedules tested in randomised studies involve a protracted course of irradiation usually to a dose of 60 Gy in 6 weeks. The treatment is given in doses $\leqslant 2$ Gy per fraction to avoid late normal tissue damage to the central nervous system (CNS), which is highly fractionation dependent.

While in terms of survival a radiotherapy treatment schedule of 60 Gy in 6 weeks is considered optimal (Chang *et al.*, 1983; Bleehen *et al.*, 1991), the overall survival of patients with high-grade glioma remains poor, and the purpose of such a protracted high-dose irradiation schedule is largely palliative. In patients destined to survive less than 6 months, who constitute 30% of high-grade glioma patients in an average cohort, 6 weeks' treatment represents 25% or more of remaining life, and this may not be acceptable to patients, relatives and physicians.

Treatment time can be shortened without reducing the biologically effective tumour dose either by reducing the number of fractions and increasing the dose per fraction (hypofractionation) or by giving the same number of fractions treating more than once a day (accelerated fractionation). The former approach may lead to an increased risk of late normal tissue damage to the brain unless the total radiation dose is reduced. The latter regimen in which multiple fractions are given each day at conventional dose per fraction, is also not without disadvantages. Repair of radiation damage in normal tissue may be incomplete in the short time interval between fractions (Ang *et al.*, 1992), and this may increase the risk of normal tissue toxicity. The logistics of twice-daily treatment also imposes strain on the normal functioning of the radiotherapy department and requires assigned machine treatment time at the two ends of the day. In addition, patients have to live close to the treatment facility or may require admission or day care to be able to attend in the early morning and late afternoon. Nevertheless, some would consider it a major advantage to complete treatment in 3 rather than 6 weeks.

We set out to examine the efficacy and toxicity of accelerated twice-daily radiotherapy in a prospective single-arm study. The results were compared with a matched cohort of patients treated with conventional daily irradiation selected from the MRC BR2 study (Bleehen *et al.*, 1991) on the basis of known prognostic factors (Table I).

### Patients and methods

Between August 1988 and June 1993, 116 patients with high-grade glioma (Table I) were treated with accelerated radiotherapy at The Royal Marsden Hospital. Seventy-four were men and 47 women, aged 19-77 years (median 56 years). Thirty-two patients had grade III and 44 grade IV astrocytoma (Kernohan and Sayre, 1952). Thirty-nine patients had high-grade tumours not otherwise specified. The presurgical Karnofsky performance status ranged from 10 to 100 (median 90) and preradiotherapy status was 40-100 (median 90). The follow-up of surviving patients ranged from 2 months to 37 months (median 9 months).

Before radiotherapy seven patients had apparent complete macroscopic tumour removal, 62 partial removal and 46 biopsy alone (one unknown extent). Patients gave informed consent to receive accelerated treatment. The tumour extent was defined on contrast-enhanced preoperative CT and/or MR images. All patients received planned radiotherapy to a target volume defined as the region of enhancement on pre operative CT and/or MRI scans plus a 3 cm margin. In unenhancing tumours a 2-3 cm margin was added to the region of abnormality. The dose to the target volume was prescribed to 100% (normalised to the point of intersection of beams). The maximum target volume inhomogeneity was

Correspondence: M Brada
Received 15 November 1994; revised 19 December 1994; accepted 10 January 1995

Table I Patient characteristics and survival

| Characteristic | Patients | Deaths | Survival (%) at 1 year | Survival (%) at 2 years | Significance |
|---|---|---|---|---|---|
| All patients | 116 | 92 | 33 | 13 | |
| Sex | | | | | NS |
| Male | 74 | 59 | 29 | 14 | |
| Female | 42 | 33 | 41 | 17 | |
| Age (years) | | | | | $P<0.005$ |
| $<55$ | 53 | 39 | 46 | 29 | |
| $\geqslant 55$ | 63 | 53 | 22 | 3 | |
| KPS before radiotherapy | | | | | $P<0.01$ |
| $\leqslant 70$ | 17 | 16 | 6 | 0 | |
| $>70$ | 99 | 76 | 38 | 18 | |
| Grade | | | | | NS |
| III | 32 | 26 | 43 | 30 | |
| IV | 44 | 39 | 26 | 8 | |
| Unspecified | 39 | | | | |
| Surgery | | | | | NS |
| None/biopsy | 46 | 36 | 25 | 12 | |
| Partial | 62 | 49 | 41 | 16 | |
| Complete | 7 | 6 | 17 | 17 | |
| Fits | | | | | NS |
| None | 77 | 61 | 35 | 15 | |
| History<3 months | 19 | 19 | 29 | 16 | |
| History $\geqslant$3 months | 12 | 12 | 32 | 11 | |

KPS, Karnofsky performance status; NS, not statistically significant.

10%. Patients were treated on a 5 or 6 MV linear accelerator with two or three fields of irradiation as defined on treatment planning. Radiotherapy was given twice a day with a minimum 6 h gap between fractions to a dose of 55 Gy. Sixteen patients received 32, 57 patients 34 and 38 patients 36 fractions (including 2 in 35 and 2 in 37 fractions), at 1.72, 1.62 or 1.53 Gy dose per fraction respectively. Five patients did not complete radiotherapy: two died before completion and three suffered progressive neurological deterioration and treatment was discontinued. No adjuvant or neoadjuvant therapy was given.

The clinical assessment of functional status was performed using a Karnofsky performance index and WHO score, and in the later part of the study patients were assessed using a modified verbally administered Barthel index (Laing *et al.*, 1993). The treatment at the time of relapse was individualised: 15 patients were treated with nitrosourea-containing chemotherapy, ten patients received Temozolomide and three patients were entered into the stereotactic radiotherapy programme. A further two patients had stereotactic radiotherapy after failing to respond to chemotherapy.

Survival was calculated from the date of diagnosis by the Kaplan–Meier method. The Cox's proportional hazards model was used to define independent prognostic factors (Cox, 1972).

### *Matched controls*

Control patients were identified from the group of patients allocated (but not necessarily completing) a 60 Gy schedule within the MRC BR2 trial (MRC Brain Tumour Working Party, 1990). This trial compared 45 Gy given in 20 fractions over 4 weeks with 60 Gy in 30 fractions. In the latter schedule the initial 40 Gy was given to a volume that encompassed all known and potential tumour followed by 20 Gy to a reduced target volume to encompass the defined tumour with a 1 cm margin.

Using prognostic factors identified from a previous MRC trial (MRC Brain Tumour Working Party, 1990), an attempt was made to match each accelerated radiotherapy patient with a control from the MRC BR2 cohort. Thus controls were of the same age ± 5 years (in practice most were of identical age), with a similar history of fits (none, less than 3 months from diagnosis or more than 3 months from diagnosis), the same preradiotherapy WHO performance status and the same extent of previous neurosurgery (biopsy, partial resection, complete resection).

The end point used to compare patients and their matched controls was survival time, dated from the start of radiotherapy. Survival curves were calculated using the Kaplan–Meier method, and compared using the log-rank test (Peto *et al.*, 1977). The hazard ratio was used as an estimate of the ratio of median survival times.

### Results

A total of 116 patients with high-grade glioma were treated with accelerated radiotherapy between 1988 and 1993. A total of 111 patients completed the planned treatment. The median survival of the whole cohort was 10 months with 33% surviving 1 year, 13% surviving 2 years and 5% surviving 3 years (Table I and Figure 1).

Age and preradiotherapy performance status were significant independent prognostic factors for survival on multivariate analysis (Table I). Gender, histological grade, the extent of surgery and previous history of seizures were not of prognostic significance.

**Figure 1** Actuarial survival of 116 patients with high-grade glioma treated with accelerated radiotherapy.

Following the start of accelerated radiotherapy, seven patients had a transient deterioration in performance status without tumour progression – six during treatment and one 11 weeks after the start. The duration of deterioration ranged from 3 to 8 weeks and subsequent improvement was maintained for an average of 30 weeks. Three patients returned to their previous status and four did not fully recover neurologically. Four patients required an interruption in radiotherapy owing to the transient deterioration. Fifteen further patients began to decline during radiotherapy owing to presumed tumour progression and continued to deteriorate without recovery. Four did not complete the course of treatment. The remaining 83 patients improved or remained stable throughout treatment.

*Comparison with matched controls*

Matched controls could be found only for 101 patients treated with accelerated radiotherapy (Table II). Their mean age was 52.6 years (s.d. 10) and for the matched controls 52.9 years (s.d. 10).

Three of the control patients remain alive with follow-up of 407, 687 and 1577 days; 14 patients treated with accelerated radiotherapy are alive with follow-up between 42 and 1180 days (median 354 days). Because of the difference in length of follow-up, two analyses were performed. The first included all 101 study patients and their matched controls. The survival comparison is shown in Table III and Figure 2. There is a suggestion of a slightly different survival pattern, with the study patients having a higher death rate in the first 12 months; however, the survival curves beyond this point are similar, and the overall log-rank comparison gave a $P$-value of 0.39. The hazard ratio was 1.13, with 95% confidence limits 0.85–1.51. In the second analysis, only those patients treated before the end of 1992 were included (84 patients), together with their matched controls. The results were very similar, with a hazard ratio of 1.12 (95% CI 0.8–1.53).

### Discussion

Radiotherapy remains the most effective treatment modality in patients with high-grade glioma (Brada, 1989), although the results of treatment remain poor with median survival less than 1 year. Little success has been achieved with attempts to improve results using radiosensitisers, hyperbaric oxygen, neutron beam therapy or altered fractionation. Although radiotherapy is largely a palliative treatment aiming to improve quality of life as well as prolonging survival, the total dose required for optimum tumour control is in the region of 60 Gy, equivalent to a radical course of radiotherapy. A randomised study comparing 45 Gy in 20 fractions with 60 Gy in 30 fractions has shown a survival advantage for patients treated with 60 Gy (Bleehen *et al.*, 1991), while in a randomised study the addition of a 10 Gy boost following 60 Gy to the whole brain (to a total tumour dose of 70 Gy) did not prolong survival further (Chang *et al.*, 1983). Conventional radiotherapy given daily over 6 weeks may therefore be considered optimal for large-volume fractionated external beam radiotherapy, but in the light of the poor overall survival the length of treatment may not be acceptable to patients or their carers.

The duration of radiotherapy can be reduced by hypofractionation or accelerated fractionation. Hypofractionated radiotherapy, giving an identical total dose of radiation at larger doses per fraction, is considered to carry an unacceptable risk of late normal tissue damage. Tested in a conservative form as 45 Gy in 20 fractions of 2.5 Gy per fraction, the survival results also appear to be inferior to conventional full dose irradiation (Bleehen *et al.*, 1991). The question remains whether full dose radiation can be given over a shorter period of time using accelerated radiotherapy.

The traditional radiotherapy schedule of 60 Gy in 30 daily fractions was modified to an accelerated schedule of 55 Gy in 34 fractions on the basis of radiobiological considerations. The repair of radiation damage in the CNS may not be completed in the 6 hour interval between fractions (Ang *et al.*, 1992). Clinical studies of continuous hyperfractionated accelerated radiotherapy (CHART) in which the interfraction interval was reduced to 4 hours resulted in an unexpectedly high incidence of myelopathy (Dische *et al.*, 1988; Dische, 1991) most likely because of incomplete repair between fractions. Experimental evidence would suggest 16% reduction in isoeffect dose for myelopathy when the inter-

**Table II** Characteristics of 101 patients treated with accelerated radiotherapy and matched with controls from MRC BR2 trial

| *Characteristic Group* | *Number of patients* |
|---|---|
| WHO performance status | |
| 0–1 | 71 |
| 2 | 26 |
| 3–4 | 4 |
| Extent of surgery | |
| Biopsy | 42 |
| Partial removal | 53 |
| Complete removal | 6 |
| Fits | |
| None | 66 |
| History <3 months | 23 |
| History ⩾3 months | 12 |

**Figure 2** Survival comparison of 101 patients with high-grade glioma treated with accelerated radiotherapy and 101 matched control patients treated with conventional daily radiotherapy in an MRC BR2 trial. Survival was measured from the start of radiation therapy.

**Table III** Survival comparison of patients treated with accelerated radiotherapy and matched controls receiving daily fractionation

| *Treatment* | *Number of patients* | *Number of deaths Observed* | *Number of deaths Expected* | *Hazard ratio (95%CI)* | *Log-rank* $\chi^2$ | *Log-rank* P |
|---|---|---|---|---|---|---|
| Accelerated radiotherapy (study) | 101 | 82 | 78 | 1.13 (0.85–1.51) | 0.73 | 0.39 |
| Conventional radiotherapy (control) | 101 | 91 | 95 | | | |

Table IV Radiotherapy studies of hyperfracted and/or accelerated radiotherapy

| Study authors | Study design[a] | No.of patients | Dose/fraction (Gy) | Treatments /day | Total dose[b] (Gy) | Treatment time (weeks) | Survival advantage |
|---|---|---|---|---|---|---|---|
| Douglas *et al.* (1982) | N | 30 | 1.0 | 3 | 45–60 + 10B | 5 | Yes |
| Payne *et al.* (1982) | R | 157 | 1.0 | 4 | 36–40 | 2 | No |
| Shin *et al.* (1983) | R | 69 | 0.89 | 3 | 40 + 10B | 4 | Yes |
| Fulton *et al.* (1984) | R | ? | 0.89 | 3 | 61.4 | 4.5 | No |
| Keim *et al.* (1987) | N | 47 | 1.6 | 3 | 60 | 2 | No |
| Ludgate *et al.* (1988) | R | 76 | 0.76 | 3 | 40 *vs* 47.6 + 10B | 5 | No |
| Deutsch *et al.* (1989) | R | 603 | 1.1 | 3 | 66 | 6 | No |
| Hernandez *et al.* (1990) | N | 14 | 1.0 | 3 | 55 | 3 | No |
| Goffman *et al.* (1992) | N | 45 | 1.5 | 2 | 70–75 | 5 | No |
| Curran *et al.* (1992) | R | 304 | 1.6 | 2 | 48 *vs* 54.4 | 3.5 | No |
| Nelson *et al.* (1993) | R | 435 | 1.2 | 2 | 64.8–72 *vs* 76.8 | 6 | No |
| | R | | 1.2 | 2 | 72 *vs* 81.6 | 6 | No |

[a]R, randomised; N, non-randomised. [b]B, boost.

fraction interval is reduced from 24 to 8 h (Ang *et al.*, 1992). The accelerated schedule was therefore modified to a total dose of 55 Gy which was sequentially given in 36, 34 and 32 fractions.

The overall results of this approach with a median survival of 10 months are similar to those of other large series. There was also no significant difference in survival between accelerated radiotherapy patients and controls matched for major prognostic factors who had 60 Gy in 30 daily fractions in a previous MRC study. While the results suggest equivalent efficacy in terms of survival, this is not a randomised study, and it is possible that patients receiving accelerated radiotherapy and the control group are unbalanced with respect to some unknown prognostic factor. The *P*-value (Table III) cannot therefore have the same meaning as a randomised comparison. Assuming exponential survival times with the hazard ratio used as an estimate of the ratio of median survival times (Table III), the value of 1.13 corresponds to an estimated increase in median survival time in control patients of approximately 5 weeks. However, the 95% confidence limits do not exclude a 7 week lengthening of median survival.

If repopulation during a protracted course of radiotherapy is an important factor determining relapse of high-grade glioma, then accelerated radiotherapy might be expected to improve survival. Failure to demonstrate an improvement does not exclude the possibility that repopulation contributes to poor results following radiation, but suggests that it is less important than other parameters determining outcome.

It is feared that accelerated radiotherapy could increase acute morbidity as well as reduce survival by increasing mortality due to early and late CNS damage. While there was no evidence of increased mortality, acute CNS toxicity is difficult to measure. During treatment six patients had a transient deterioration in performance status and one patient had a transient (3 week) deterioration after 11 weeks. Such transient effects could have been either treatment related with subsequent recovery or due to tumour progression with delayed response to treatment. There is no information on the incidence of similar events in patients treated conventionally. In our experience this type of morbidity is similar, although an increased risk of early neurological impairment cannot be excluded on the basis of these data. Accelerated hyperfractionated radiotherapy to a dose of 54.4 Gy was compared with 48 Gy (both at 1.6 Gy per fraction twice daily) in a RTOG study 83-02 with similar early toxicity and survival results (Curran *et al.*, 1992).

Fifteen patients had a gradual continuous decline in performance status which began during radiotherapy. It is difficult to separate any possible treatment-related deterioration from progressive unresponsive disease. Theoretically, deterioration due to incomplete repair and subsequent necrosis of normal tissue would not be manifest until weeks or months after the end of treatment.

Other groups have used altered fractionation in an attempt to improve survival results (Table IV). Only two small studies of hyperfractionation have shown a possible benefit (Douglas and Worth 1982; Shin *et al.*, 1983). The majority of studies did not demonstrate prolongation of survival (Table IV) and overall have little advantage compared with conventional fractionation. Increasing the total dose using hyperfractionation has also been tested. Patients treated in a RTOG trial 83-02 testing hyperfractionated accelerated radiotherapy to 54.4 Gy had a median survival of 10.8 months with little early toxicity (Curran *et al.*, 1992). In a randomised dose-searching phase I/II study of the RTOG (Nelson *et al.*, 1993), patients received doses of 1.2 Gy per fraction twice daily. The total doses ranged from 64.8 Gy to 81.6 Gy (four dose levels) and treatment was given over a period of 5.5–6.5 weeks together with adjuvant BCNU. There was no survival benefit with higher radiation doses and even a suggestion of worse results with doses $\geqslant$ 74.5 Gy. Overall accelerated and/or hyperfractionated radiotherapy is therefore of little additional survival benefit.

We conclude that in patients with high-grade glioma radiotherapy treatment time can be reduced from 6 to just over 3 weeks without a marked increase in toxicity or loss of survival benefit. The short overall survival in these patients precludes any definite conclusion about the long-term safety of high-dose accelerated irradiation. There are logistic problems in this approach which require reorganisation of treatment machine time and occasionally provision of day care or in-patient facilities for patients unable to attend twice daily. This and the overall higher number of fractions have clear financial implications for the service. Nevertheless, in our experience accelerated radiotherapy is feasible and acceptable to patients, staff and carers and has become an available treatment for selected patients who wish to complete therapy in a shorter time.

**Acknowledgements**

We are grateful to our neurosurgical colleagues at the Atkinson Morley's Hospital (Mr D Uttley, Mr H Marsh, Professor A Bell and Miss A Moore) for their collaboration and to the staff of the Radiotherapy Department at the Royal Marsden Hospital for carrying out the treatment. The medical and nursing staff of the Neuro-oncology Unit provided the care and support during and after treatment. Miss Christine Evans kindly helped in the preparation of the manuscript. The work was supported by grants from the Cancer Research Campaign, the Julian Bloom Research Fund and the Royal Marsden NHS Trust.

### References

ANG KK, JIANG GL, GUTTENBERGER R, THAMES HD, STEPHENS LC, SMITH CD AND FENG Y. (1992). Impact of spinal cord repair kinetics on the practice of altered fractionation schedules. *Radiother. Oncol.*, **25**, 287–294.

BLEEHEN NM AND STENNING SP. ON BEHALF OF THE MEDICAL RESEARCH COUNCIL BRAIN TUMOUR WORKING PARTY. (1991). A Medical Research Council trial of two radiotherapy doses in the treatment of grades 3 and 4 astrocytoma. *Br. J. Cancer*, **64**, 769–774.

BRADA M. (1989). Back to the future – radiotherapy in high grade gliomas. *Br. J. Cancer*, **60**, 1–4.

CHANG CH, HORTON J, SCHOENFELD D, SALAZAR O, PEREZ-TAMAYO R, KRAMER S, WEINSTEIN A, NELSON JS AND TSUKADA Y. (1983). Comparison of postoperative radiotherapy and combined postoperative radiotherapy and chemotherapy in the multidisciplinary management of malignant gliomas. *Cancer*, **52**, 997–1007.

COX DR. (1972). Regression models and life tables. *J.R. Stat. Soc., B*, **34**, 187–202.

CURRAN WJ, SCOTT CB, NELSON JS, WEINSTEIN AS, PHILLIPS TL, MURRAY K, FISCHBACH AJ, YAKAR D, SCHWADE JG, POWLIS WD AND NELSON DF. (1992). A randomized trial of accelerated hyperfractionated radiation therapy and bis-chlorethyl nitrosourea for malignant glioma. *Cancer*, **70**, 2909–2917.

DEUTSCH M, GREEN SB, STRIKE TA, BURGER PC, ROBERTSON JT, SELKER RG, SHAPIRO WR, MEALEY JJ, RANSOHOFF J, PAOLETTI P, SMITH KR, ODOM GL, HUNT WE, YOUNG B, ALEXANDER E, WALKER MD AND PISTENMAA DA. (1989). Results of a randomised trial comparing BCNU plus radiotherapy, streptozotocin plus radiotherapy, BCNU plus hyperfractionated radiotherapy, and BCNU following misonidazole plus radiotherapy in the postoperative treatment of malignant glioma. *Int. J. Radiat. Oncol. Biol. Phys.*, **16**, 1389–1396.

DISCHE S. (1991). Accelerated treatment and radiation myelitis. *Radiother. Oncol.*, **20**, 1–2.

DISCHE S, WARBURTON MF AND SAUNDERS MI. (1988). Radiation myelitis and survival in the radiotherapy of lung cancer. *Int. J. Radiat. Oncol. Biol. Phys.*, **15**, 75–81.

DOUGLAS BG AND WORTH AJ. (1982). Superfractionation in glioblastoma multiforme – results of a phase II study. *Int. J. Radiat. Oncol. Biol. Phys.*, **8**, 1787–1794.

FULTON DS, URTASUN RC, SHIN KH, GEGGIE PHS, THOMAS M, MULLER PJ, MOODY J, TANASICHUK H, MIELKE B, JOHNSON E AND CURRY B. (1984). Misonidazole combined with hyperfractionation in the management of malignant glioma. *Int. J. Radiat. Oncol. Biol. Phys.*, **10**, 1709–1712.

GOFFMAN TE, DACHOWSKI LJ, BOBO H, OLDFIELD EH, STEINBERG SM, COOK J, MITCHELL JB, KATZ D, SMITH R AND GLASTEIN E. (1992). Long-term follow-up of National Cancer Institute phase I/II study of glioblastoma multiforme treated with iododeoxyuridine and hyperfractionated irradiation. *J. Clin. Oncol.*, **10**, 264–268.

HERNANDEZ JC, MARUYAMA Y, YAES R AND CHIN HW. (1990). Accelerated fractionation radiotherapy for hospitalised glioblastoma multiforme patients with poor prognostic factors. *J. Neuro-Oncol.*, **9**, 41–45.

KEIM H, POTTHOFF PC, SCHMIDT K, SCHIEBUSCH M, NEISS A AND TROTT KR. (1987). Survival and quality of life after continuous accelerated radiotherapy of glioblastomas. *Radiother. Oncol.*, **9**, 21–26.

KERNOHAN J AND SAYRE G. (1952). Tumors of the central nervous system - astrocytomas. In *Atlas of Tumor Pathology*, section 10, fascicle 35. Firminger HI (ed.) pp. 313–332. Armed Forces Institute of Pathology: Washington DC.

LAING RW, WARRINGTON AP, GRAHAM J, BRITTON J, HINES F AND BRADA M. (1993). Efficacy and toxicity of fractionated stereotactic radiotherapy in the treatment of recurrent gliomas (phase I/II study). *Radiother. Oncol.*, **27**, 22–39.

LUDGATE CM, DOUGLAS BG, DIXON PF, STEINBOK P, JACKSON SM AND GOODMAN GB. (1988). Superfractionated radiotherapy in grade III, IV intracranial gliomas. *Int. J. Radiat. Oncol. Biol. Phys.*, **15**, 1091–1095.

MRC BRAIN TUMOUR WORKING PARTY. (1990). Prognostic factors for high-grade malignant glioma: development of a prognostic index. *J. Neuro-Oncol.*, **9**, 47–55.

NELSON DF, CURRAN WJ, SCOTT C, NELSON JS, WEINSTEIN AS, AHMAD K, CONSTINE LS, MURRAY K, POWLIS WD, MOHIUDDIN M AND FISCHBACH J. (1993). Hyperfractionated radiation therapy and bis-chlorethyl nitrosourea in the treatment of malignant glioma – possible advantage observed at 72.0 Gy in 1.2 Gy BID fractions: report of the radiation therapy oncology group protocol 8302. *Int. J. Radiat. Oncol. Biol. Phys.*, **25**, 193–207.

PAYNE DG, SIMPSON WJ, KEEN C AND PLATTS ME. (1982). Malignant astrocytoma. Hyperfractionated and standard radiotherapy with chemotherapy in a randomised prospective clinical trial. *Cancer*, **50**, 2301–2306.

PETO R, PIKE M, ARMITAGE P, BRESLOW N, COX D, HOWARD S, MANTEL N, MCPHERSON K, PETO J AND SMITH P. (1977). Design and analysis of randomised clinical trials requiring prolonged observation of each patient. 2. Analysis and examples. *Br. J. Cancer*, **35**, 1–39.

SHIN KH, MULLER PJ AND GEGGIE HP. (1983). Superfractionation radiation therapy in the treatment of malignant astrocytomas. *Cancer*, **52**, 2040–2043.